# Medical Life in London

**Published:** 1852-11

**Authors:** 


### EDITORIAL DEPARTMENT.

*Medical life in London.*—We have had occasion heretofore to allude to the habit, which has prevailed of late years in this country, among writers in medical journals and speakers at medical gatherings, of disparaging the moral and educational standing of American practitioners. The ranks of the profession, it is said, are filled by persons poorly educated; our medical schools are spoken of with contempt; the tendency to quackery, both within and without the profession, is a constant theme of querulous complaint. In all these particulars a comparison with other countries, of American medicine, is frequently drawn, in which the latter is made to appear greatly inferior, This habit of self-depreciation has become so confirmed, that whatever may be the progress made on this side of the Atlantic, many, it is to be feared, under the influence of ideas that have been so much reiterated, will never have courage enough to feel a national pride in whatever may be accomplished. A disposition to undervalue, in anticipation, any discovery, or improvement, originated in this country, is often apparent. An extract from a foreign journal containing something new proposed by some one wholly unknown at this distance, not infrequently passes current, or attracts attention, while a novelty, from a responsible source, which labors under the disadvantage of being a home production, may not only be overlooked, but, as it would seem, studiously kept out of sight. For example, a practitioner of long experience and high standing, announces a new method of reducing dislocations of the hip joint, without the aid of pulleys, etc. He adduces several cases in which the plan has been successfully tried. It excites some attention, but chiefly by those who are anxious to prove that the author borrowed the idea of some one else. By some journalists, and surgical teachers, the subject meets with no notice whatever. They are not willing even to make trial of the plan, and considerable effort is necessary to bring it sufficiently before the profession, to secure a sufficient number of cases for a fair experimental test of its merits. Even the great discovery of the employment of anæsthetic agents in surgery, has met with an active opposition at home, which it has not had to encounter abroad.

With the low estimation of American medicine which appears to be cherished to a considerable extent among ourselves, it may contribute somewhat to a higher grade of self-respect, to read what the profession of other countries say, not of us, but of themselves. With this view, we quote a few paragraphs from a series of articles which have lately appeared in the *Dublin Medical Press*, headed, "Medical Life in London." Speaking of medical students the writer says:

"We said enough before, perhaps, as to the very insufficient education that medical students receive before they join the classes; it is not difficult now to perceive how likely they are to be misguided as to the true and noble calling in which they are embarked by the ridiculous books and essays which fall in their way, the product of the overgrown trade in these commodities. Accordingly, one meets them constantly at Guy's, at King's, and Bartholomew's, arguing, even with the chief men there, as to their 'convictions on mesmerism,' the marvelous cures they 'had read by homœopathy.' The trite creed of too many of these young men, that all 'physic is humbug'—*their* knowledge of physic no doubt being very nonsensical—is due to the utterly absurd books they had been reading, the result of this frightful trading principle of the book-trade, and the favoritism shown to special authors who happen to have money. * * * * * * In London, the student sees the uneducated chemist and the College of Surgeons the only people making fortunes. He votes physic a bore; the College of Physicians, like the Court of Chancery, a great professional incubus to be avoided; advancement in professional life as impossible as the discovery on his own private account of perpetual motion. He knows half the money he has lost would get him a commission in the army, or set him up in a cotton-mill. He has heard of Apothecaries' Hall; but on looking into the books he thinks he might as well try to learn Dutch and Sanscrit, as Dr. Lindley's big words; he never does learn them, for he never learned Greek or Latin; he has been reading novels and the book about Egypt, and the salt-cellars, or the latest rubbish sent 'with the author's compliments' to his hospital library. Three years, four, and sometimes five, he spends in this mortifying way. In October, he comes up, like all his fellow-geese, poor fellow, to be plucked, and to hear the introductory lectures, at which he is told his profession is all pleasantness and all its paths peace, that he has only to follow the directions of each particular lecturer to make a solid fortune, and gain a commanding corner in the Temple of Fame.

If now he runs away without diploma or certificate, and sets up a chemist's shop, he is safe; if he waits for academic honors and College of Surgeons' *soirées*, he will rue it all the days of his life. Godfrey's cordial and chemicals carry the day; or if the trade in homœopathicals promises better, he has no scruples, for long since he has decided 'physic all humbug.' Even Dr. Pereira, who is considered a Jew, and who, one would think, should make money, if any body did, out of medicine, is of opinion all English physic is nonsensical."

Of the profession he adds:

"We have just had a meeting of the Provincial Medical Association at Oxford; but with such a disjointed and dissociated mass as the profession in London, one looks in vain for anything very enlivening at these gatherings. If one could read in the signs of the times, or in any point of the professional zodiac, of a complete sweeping away of all present overgrown abuses, then might one indeed breathe freely the open air of Heaven. Like the reform in its next-door neighbor Chancery, brought about by the pen of honest, thoughtful men like Dickens, perhaps other parts of Lincoln's-inn-fields will yet undergo some change for the better; and when the pepper-boxes of the National Gallery at the West-end of London, the not very captivating or odorous abuses of the College of Physicians also next door, and two or three quack hospitals, are also removed, we may yet sing pæans of thanksgivings to what Mr. D'Israeli calls the 'spirit of the age.' A member of the College of Physicians of Dr. Paris, may be sent to Newgate if he perpetrates the crime of consulting with the president, or any member, of any Dublin or Edinburgh College of Physicians, or any other M. D. 'whatsomnever;' but they have been known to send nice three-cornered notes, appointing nice three-cornered hours to meet fashionable homœopaths. At the *soirées*, also, one sees emblazoned in all the papers, the sorriest kind of tuft-hunting is had recourse to, and strangers of Rome, 'Cretes and Arabians,' any body and every body, but exactly those for whom one would think royal colleges, medical and chirurgical, were erected, are invited; the pleasant ultimate result of all such corporate bodies here being that all struggling medical men might as well have a millstone around their neck as the excruciatingly absurd care and patronage of these big buildings. With money, of course, young medical men in London, will make a fortune. A slender apprenticeship to quackery, however, is as indispensable as kid gloves at the College of Physicians' tea parties. Any man in Ireland with £1000 to purchase a practice or partnership, with energy and ability to work, in the present rush of surgeons to the gold diggings, would make a very favorable percentage on the outlay, and bring up his family respectably. Let him think, only, of the presidents of the two colleges, as fabulous people, like Mopsus and Corydon; his diplomas as so much waste paper; the Hunterian oration every year as some blissful condition of things in the book of the Crusades; the medical weekly journals not so much paladins as pump-borers, pumping every man they can, and then throwing him one side as useless. If the man wishes to be happy and contented, and live among his patients, he will sedulously avoid all and each of these. If he is a quack, it is painful to repeat again, he is sure of a fortune. If he is honest, the millstone of the journals and colleges will be his destruction."

Again:

"The practice of physic and surgery in the hospitals is unexceptionable. The moral influence of the colleges and press out of doors, the most melancholy sham; quackery and trade existing in every department of the profession; but perhaps the lowest and the highest, in the court and highest circles, and in the daily drudgery of the lowest or union practice among the poor, the experience of every disinterested man is against quackery; but the homœopathist or the traveling charlatan, when he makes money enough of his wares, goes up to the College of Surgeons and gets his diploma; the chemist boldly advertises for a medical student to prescribe over the counter; the College of physicians makes it impossible for any but such juveniles to make money, and they and the chemists wax fat on the credulity of the people. Cunning and incapableness, coroners' inquests, and Hunterian orations, white kid gloves, and flunkeyism, all going to make up the staple—the web and woof of English medical practice in this happy year of our Lord, 1852."

We cannot, of course, vouch for the correctness of the representation of medical life in London, as given in the above quotations. With respect to this point it is not to be forgotten that London and Dublin are different places, although situated in Great Britain. How much of the spirit of the articles referred to is due to rivalry of location, we cannot presume to say. We have given the quotations in order that those of our readers who would be glad to think better of medical life in their own country than they who appear to have a fondness for disparaging it, may be encouraged by the fact that in the great English metropolis, a writer on the spot finds as much scope for animadversion and ridicule, as the warmest advocate of American inferiority could claim in behalf of the medical profession on this side the Atlantic.

